# Non-targeted analytical comparison of a heated tobacco product aerosol against mainstream cigarette smoke: does heating tobacco produce an inherently different set of aerosol constituents?

**DOI:** 10.1007/s00216-024-05126-x

**Published:** 2024-01-13

**Authors:** Gerhard Lang, Carlos Henao, Martin Almstetter, Daniel Arndt, Catherine Goujon, Serge Maeder

**Affiliations:** PMI R&D, Philip Morris Products S.A, Quai Jeanrenaud 5, CH-2000 Neuchâtel, Switzerland

**Keywords:** Heated tobacco product, Cigarette smoke, Untargeted analysis, Gas chromatography, High-performance liquid chromatography, Mass spectrometry

## Abstract

Smoking-related diseases remain a significant public health concern, and heated tobacco products (HTPs) have emerged as a potential alternative to cigarettes. While several studies have confirmed that HTP aerosols contain lower levels of harmful and potentially harmful constituents (HPHCs) than cigarette smoke, less is known about constituents that are intrinsically higher in HTP aerosols. This study provides a comprehensive comparative assessment of an HTP aerosol produced with Tobacco Heating System 2.2 (THS) and comparator cigarette (CC) smoke aiming at identifying all unique or increased compounds in THS aerosol by applying a broad set of LC–MS and GC × GC–MS methods. To focus on differences due to heating versus burning tobacco, confounding factors were minimized by using the same tobacco in both test items and not adding flavorants. Of all analytical features, only 3.5%—corresponding to 31 distinctive compounds—were significantly more abundant in THS aerosol than in CC smoke. A notable subset of these compounds was identified as reaction products of glycerol. The only compound unique to THS aerosol was traced back to its presence in a non-tobacco material in the test item and not a direct product of heating tobacco. Our results demonstrate that heating a glycerol-containing tobacco substrate to the temperatures applied in THS does not introduce new compounds in the resulting aerosol compared to CC smoke which are detectable with the method portfolio applied in this study. Overall, this study contributes to a better understanding of the chemical composition of HTP aerosols and their potential impact on human health.

**Graphical Abstract:**

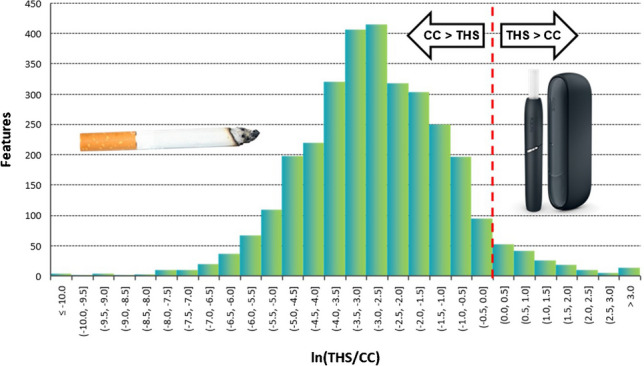

**Supplementary Information:**

The online version contains supplementary material available at 10.1007/s00216-024-05126-x.

## Introduction

Heated tobacco products (HTPs) have gained popularity as an alternative to cigarettes and are now commercially available in many countries worldwide. Also known as heat-not-burn products, HTPs differ from cigarettes in that they heat tobacco rather than burn it, thus producing a nicotine-containing aerosol with lower chemical complexity than cigarette smoke. HTPs operate by heating a substrate of processed tobacco, which can also contain various additives such as flavors and glycerol, which enables the formation of a visible aerosol with agreeable sensorial properties. The tobacco is heated to a temperature that is lower than the combustion temperature in cigarettes, typically between 250 and 350 °C, which results in the release of an aerosol that contains nicotine and other chemical constituents.

The relevance of this product category lies in its potential as a harm reduction strategy, as HTPs have been shown to release lower levels of harmful and potentially harmful constituents (HPHCs) than cigarettes. For one of the market-leading HTPs, the Tobacco Heating System 2.2 (THS 2.2) developed by Philip Morris International (PMI) and marketed under the brand name *IQOS*™ [1], analytical studies have substantiated its potential for harm reduction by demonstrating that it releases on average > 90% lower levels of HPHCs versus a reference cigarette, even when using extreme puffing regimens and climatic conditions [2, 3].

However, analytical studies comparing the levels of predefined HPHCs in HTP aerosol and cigarette smoke are insufficient for determining whether new chemical hazards exist with HTPs. Specifically, they do not show whether the heating process generates other compounds of toxicological relevance that are not present or are present in lower concentrations in cigarette smoke. To address this question, untargeted analytical methods are required to detect all constituents of the HTP aerosol and provide structural information as well as (semi-)quantitative abundances of these compounds to enable an assessment of their toxicological impact.

As part of the data made available to the U.S. Food & Drug Administration (FDA) in support of the Modified Risk Tobacco Product Application (MRTPA) for the THS 2.2, PMI performed non-targeted differential screening (NTDS) studies utilizing both LC-HRAM-MS and GC × GC-TOFMS methods. These methods aim to deliver maximum coverage for the chemical space relevant to tobacco-derived aerosols (i.e., polarity and molecular weight range) and were applied to identify compounds with per-stick yields in aerosol of the three THS 2.2 variants included in the MRTPA that were higher than in smoke of the 3R4F reference cigarette used as a comparator [4]. These studies identified 85 compounds as being increased in THS aerosol compared to the reference cigarette in at least one of the tested variants [5]. A toxicological evaluation of these 85 aerosol constituents highlighted four compounds classified as carcinogens and/or mutagens (glycidol, 3-monochloro-1,2-propanediol [3-MCPD], 2-furanmethanol, and furfural), underscoring the usefulness of untargeted analytical methods for identifying previously unrecognized toxicological hazards of new products. However, the increased levels of these compounds were deemed not critical for the overall harm reduction potential of THS 2.2. The FDA’s Technical Project Lead stated that “Although some of the chemicals are genotoxic or cytotoxic, these chemicals are present in very low levels and potential effects are outweighed by the substantial decrease in the number and levels of HPHCs found in CC [combusted cigarettes].” [6]. In addition to these NTDS studies, PMI conducted a chemical characterization of THS 2.2 aerosol, providing chemical identifications for all 529 aerosol constituents with semi-quantitative per-item yields above 100 ng/item (excluding glycerin, nicotine, and water), as well as the respective semi-quantitative yields in 3R4F smoke. Only a minority of these compounds were present at concentrations exceeding those measured in the smoke of the cigarette [7]. With respect to the reduced chemical complexity of HTP aerosols when compared to 3R4F smoke, similar results were also obtained by other research groups [8–11].

While the above-cited untargeted analytical studies provided a comprehensive overview of the chemical composition of THS 2.2 aerosols and an evaluation of which constituents are present only in THS 2.2 aerosol or are more abundant than in reference cigarette smoke, they do not allow for an unequivocal conclusion on which of the observed differences are an implicit characteristic of the THS platform (i.e., the effect of heating instead of burning tobacco). The question remains whether the compounds unique to THS 2.2 aerosol are an inherent consequence of heating the tobacco substrate or an artifact of the experimental design. The most important confounding factor to be highlighted for these studies is that the tobacco substrate heated in THS 2.2 differed in several important aspects from the tobacco in the reference cigarette:

- The tobacco substrate in the THS 2.2 test items contained added flavor ingredients, which can transfer to the aerosol, while the 3R4F reference cigarette is unflavored [4].
- The 3R4F cigarette contains 6.41% added sugar (Isosweet) [4], but the THS 2.2 substrate has no sugar added.
- The tobacco blend structure (i.e., the composition by tobacco types and their percentage of inclusion in the blend) of 3R4F reference cigarettes is different from the typical blend structure used in THS 2.2.

It is known that different tobacco additives can influence an untargeted differential screening, but also tobacco blend structure has been shown to be a crucial factor in determining the resulting chemistry of HTP aerosols and cigarette smoke. The blend in the 3R4F cigarette is a typical American Blend with > 21% Burley tobacco [4], which is rich in nitrogenous compounds, e.g., proteins, amino acids, and organic acids. In comparison, the blends used in THS 2.2 contain a higher percentage of flue-cured tobacco, which has higher quantities of natural sugars but less nitrogenous compounds [12]. Significant differences exist between these two types of blends in their respective aerosol compositions whether following heating or combustion. For example, while American blended cigarettes produce more nitrogenous compounds upon combustion, flue-cured Virginia blends generate more carbonyls and sugar degradation products [13]. When used in the THS 2.2, experimental blends containing a high proportion of nitrogen-rich tobaccos produced higher yields of nitrogenous compounds (e.g., tobacco-specific nitrosamines, ammonia, nitrogen oxides, and acrylamide); therefore, for commercial THS tobacco blends, strict inclusion limits for nitrogen-rich tobacco types are applied [14]. It should be noted that even if the previous NTDS studies had been performed with test items and comparator cigarettes of the same blend structure, differences in tobacco chemical composition due to agricultural variability (e.g., climatic conditions or fertilization regimes) and the specific tobacco varieties used could have introduced aerosol chemistry differences making it difficult to pinpoint the differences due to heating vs. burning.

An unbiased and comprehensive untargeted analysis of tobacco aerosols requires capturing the greatest possible diversity of compounds in this matrix, which span numerous structural classes and exhibit varied physical properties. This is best achieved by employing both LC and GC platforms and multiple chromatographic methods covering a wide polarity and volatility range. The advent of GC × GC instrumentation led to a technology with inherently higher peak capacity than one-dimensional GC, which is ideally suited for the untargeted analysis of complex matrices as it increases the number of well-separated chromatographic peaks [15]. Mass spectrometry remains the preferred detection method for untargeted analyses due to the high information content of MS data, its sensitivity, and the availability of large databases of reference spectra. The use of high-resolution mass spectrometry (HRMS) further enhances the accuracy of compound identification.

This study aimed to identify all aerosol constituents exclusive to THS 2.2 aerosol or with higher yields in THS 2.2 aerosol than in cigarette smoke, focusing exclusively on chemical differences due to heating vs. burning tobacco. The test item design considered three points to address confounding factors: (1) the same tobacco blend structure was used for THS 2.2 and comparator cigarette test items, (2) tobacco from the same crops was used to exclude possible differences due to agricultural variability, and (3) no flavors were added to the test items. Analyses of the aerosol and smoke samples applied a similar set of GC × GC-TOFMS and LC-HRAM-MS methods as in previous studies [5, 7]. Structural proposals for all increased compounds were obtained through a combination of database searches and the prediction of chromatographic retention properties and confirmed wherever possible by the injection of authentic reference standards. In addition to the untargeted screening, nicotine and four compounds of toxicological relevance previously found to be more abundant in THS 2.2 aerosol (2-furanmethanol, furfural, glycidol, and 3-MCPD) were quantified using validated methods.

## Materials and methods

### Materials

THS 2.2 test items (THS) and comparator cigarettes (CC) were produced at Philip Morris Products SA (Neuchâtel, Switzerland). A blend of different tobacco types was prepared consisting predominantly of flue-cured tobacco (> 60%), Oriental tobacco, and a small fraction of air-cured type tobaccos (< 10%). The same blended tobacco was used for manufacturing the reconstituted tobacco sheet for the THS items and to prepare tobacco cut filler for the CC. The average masses of reconstituted tobacco in THS and of tobacco cut filler in CCs were 306 and 603 mg/item, respectively. Besides finely ground tobacco, the THS substrate contained small concentrations of guar gum and cellulose fibers to enable the manufacturing of a stable reconstituted tobacco sheet, and glycerol (17.7%) as an aerosol former. Propylene glycol (0.9% w/w), which in commercial THS 2.2 products is used as solvent when applying flavors, was sprayed onto the final tobacco sheet. Design parameters (dimensions, filter type, ventilation) of the CC were aligned to those of the 1R6F research cigarette, including the levels of added humectants (glycerol, 1.7% w/w; propylene glycol, 1.0% w/w) [16]. The design of THS 2.2 items and the system operating principles were described in a previous publication [1].

Details on the solvents and standards used for the preparation and analysis of smoke and aerosol samples are provided in the Electronic Supplementary Material (Table S1).

### Sample preparation

Prior to aerosol/smoke generation, THS consumables and CCs were conditioned according to ISO 3402 [17] for a minimum of 48 h and a maximum of 10 days at 22 ± 1 °C and 60 ± 3% relative humidity (RH). The conditioning was performed in open packages for all test items.

The Health Canada Intense (HCI) puffing regime (Health Canada, T-115, 1999) [18] was applied to generate mainstream aerosol or smoke; it is commonly used and recognized as a standard smoking protocol by experts and official organizations for the assessment of both cigarettes and heat-not-burn products. CCs were 100% vent-blocked by taping. THS items were not taped due to absence of ventilation holes in the filter region. The room conditions for aerosol generation were 22 ± 2 °C and 60 ± 5% RH. For THS, 12 puffs were collected per item, while CCs were smoked to a fixed butt length of 35 mm, achieved approximately after 10 puffs.

**Fig. 1:**
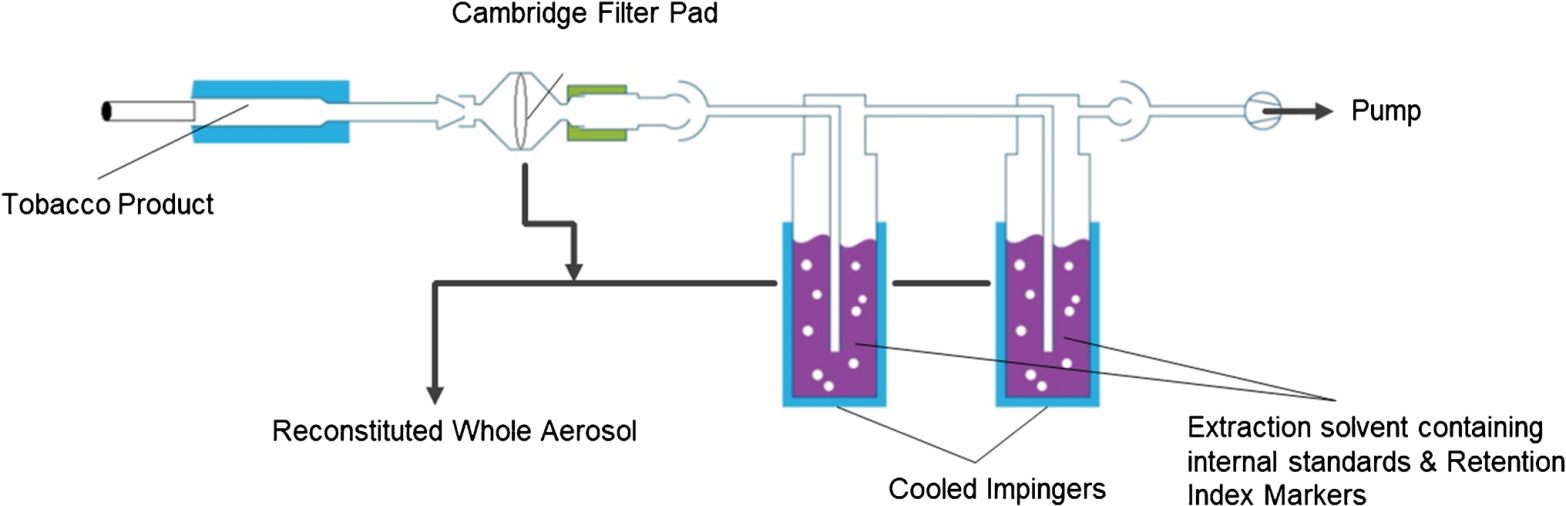
Experimental setup for aerosol/smoke trapping and the generation of reconstituted mainstream whole aerosol/smoke (adapted from Bentley et al.) [7]

Aerosol (THS) and smoke (CC) trapping were performed using a Cambridge glass fiber filter pad (CFP; diameter 44 mm) followed by two consecutive impinger traps, each filled with a solvent (10 mL; Fig. 1, Table 1) and internal standards specific to the respective analytical method (see Electronic Supplementary Material, Tables S6, S8, and S9); four different sampling conditions (i.e., trapping solvents, temperatures, standards) were applied for each test item to generate reconstituted whole aerosol/smoke samples suitable for the full set of analytical GC and LC methods. In addition, blank collections were performed with the same puffing parameters (12 puffs) but without test items. Ten replicate samples were collected for both test items and blanks. Each aerosol replicate comprised either the accumulated trapped aerosol from five THS items or smoke from three CCs. After each aerosol or smoke collection, the CFP was extracted with the combined solvent from the two impingers to generate a reconstituted aerosol/smoke solution.

**Table 1:** Method-specific trapping solvents and temperatures

Method	Trapping solvent	Trapping temperature
LC–MS RP	Methanol	− 60 °C
LC–MS HILIC	Acetonitrile	0 °C
GC × GC–MS polar/nonpolar	CH_2_Cl_2_:acetone (80:20 v/v)	− 80 °C
GC × GC–MS volatile	Dimethylformamide	− 60 °C

An aliquot (10 mL) of the solution obtained with the GC × GC–MS polar/nonpolar trapping regimen was partitioned against an equal volume of water and the resulting organic phase subjected to GC × GC–MS analysis using the nonpolar method after drying over sodium sulfate; the aqueous phase was analyzed with the polar method after addition of the method-specific internal standards (see Electronic Supplementary Material, Table S7). The solution obtained with the trapping regime optimized for the GC × GC–MS volatile method was analyzed without further work-up. For LC-HRAM-MS analyses, aliquots (300 μL) of the reconstituted aerosol/smoke solutions were diluted with methanol (700 μL) or acetonitrile (700 μL) for reversed-phase (RP) or hydrophilic interaction liquid chromatography (HILIC), respectively. Pooled samples representing the entire chemical space of the sample set were prepared for each individual analytical method by combining equal volumes of all reconstituted aerosol and smoke samples. These pooled samples were used for chromatogram alignment and generic peak finding process; they served as system suitability test and quality control samples.

### Analytical methods

Ten replicates of reconstituted whole aerosol or smoke for each test item, as well as the blank samples and pooled samples, were subjected to GC × GC-TOFMS and LC-HRAM-MS analyses. Both technologies apply a set of diverse analytical methods to ensure the coverage of a wide range of constituent polarities, volatilities, and structural classes. Each aerosol sample was injected once (i.e., no injection replications were performed). Example chromatograms for THS and CC analyzed with all analytical methods are provided in the Electronic Supplementary Material (Fig. S1–S7).

GC × GC-TOFMS analyses comprised a total of three analytical methods (GC Polar, GC-Nonpolar, and GC Volatile) covering different ranges of polarity and volatility. The analyses were performed using a Model 7890A or 8890 gas chromatograph (Agilent Technologies, Santa Clara, CA, USA) equipped with an Auto Liquid Injector (Model 7683 or 7693) and a Thermal Modulator coupled to a Pegasus® 4D TOFMS (LECO Corporation, St. Joseph, MI, USA) for nominal mass (Polar and Volatile method) and to a Pegasus® HRT + 4D for high-resolution accurate mass measurements (Nonpolar method).

#### Nonpolar method:

GC × GC separation was performed using a DB-5 ms (30 m × 0.25 mm internal diameter [ID] × 0.25-µm film thickness, Agilent Technologies) and DB-17ht (2.3 m × 0.10 mm ID × 0.10-µm film thickness, Agilent Technologies) chromatographic column combination for the first and second dimensions, respectively. Helium was used as carrier gas and kept at a constant flow of 1.0 mL/min. A linear temperature program was used, starting at 30 °C (held for 2 min) and increasing at 5 °C/min to 325 °C (15 min) for the first dimension, and starting at 35 °C (held for 2 min) and increasing at 5.2 °C/min to 340 °C (15.3 min) for the second dimension. Modulation time was set to 6 s. Ion source temperature was set at 230 °C, and the transfer line temperature was set at 300 °C. High-resolution accurate mass spectra were acquired with an electron energy of − 70 eV, with a data acquisition rate of 200 spectra per second using a scan range of 35–545 Da. The samples were injected using a cool on-column mode with an injection volume of 0.1 µL. The cool on-column injector temperature was ramped to track the oven temperature with a positive offset of 3 °C.

#### Polar method:

GC × GC separation was performed using an ionic liquid pre-column (SLB-IL60, 2 m × 0.25 mm ID × 0.20-µm film thickness, Supelco (Merck KGaA) and a DB-FFAP (2.4 m × 0.10 mm ID × 0.10-µm film thickness, Agilent Technologies) and VF-624 ms (1.9 m × 0.15 mm ID × 0.84-µm film thickness, Agilent Technologies) as first- and second-dimension columns, respectively. Helium was used as carrier gas and kept at a constant flow of 1.0 mL/min. A linear temperature program was used, starting at 35 °C (held for 2 min) and increasing at 5 °C/min to 250 °C (23 min) for the first dimension, and starting 55 °C (held for 2 min) and increasing at 4.6 °C/min to 285 °C (16 min) for the second dimension. Mass spectra were acquired at 200 Hz using a scan range of 29–700 Da. The remaining parameters were set as described for the Nonpolar method.

#### Volatile method:

GC × GC separation was performed using a DB-624UI (30 m × 0.25 mm ID × 1.40-µm film thickness, Agilent Technologies) and DB-FFAP (2.2 m × 0.10 mm ID × 0.10-µm film thickness, Agilent Technologies) as first- and second-dimension columns, respectively. The temperature program of the primary oven started at − 20 °C using an active liquid nitrogen oven cooling system (held for 1 min) and increased at 5 °C/min to − 5 °C, at 1 °C/min to 50 °C, at 5 °C/min to 95 °C, and at 45 °C/min to 230 °C (9 min). The temperature program for the secondary oven started at 0 °C (held for 4 min) and increased at 1 °C/min to 55 °C, at 5 °C/min to 100 °C, and at 45 °C/min to 235 °C (9 min). The transfer line temperature was maintained at 250 °C. Mass spectra were acquired at 200 Hz using a scan range of 29–500 Da. The remaining parameters were set as described for the Nonpolar method.

LC-HRAM-MS analyses were performed on a hybrid Thermo Scientific Q Exactive™ Plus high-resolution mass spectrometer (Thermo Fisher Scientific, Waltham, MA, USA) in conjunction to a Vanquish LC System comprising a Horizon Binary Pump, Vanquish Split Sampler HT, and a Vanquish Column Compartment (Thermo Fisher Scientific, Waltham, MA, USA). In total, four different LC–MS methods were applied: reversed-phase (RP) chromatography with heated electrospray ionization (HESI) in both positive and negative modes and with atmospheric pressure chemical ionization (APCI) in positive mode, in addition to HILIC chromatography with HESI in positive mode. For all LC–MS methods, mass spectral data was acquired in full scan mode with additional data-dependent fragmentation to enable the identification of relevant compounds [19].

#### RP-HESI(+) method:

LC separation was performed using a Hypersil GOLD™ column (150 × 2.1-mm ID, 1.9 μm; Thermo Scientific) preceded by a UHPLC guard filter cartridge (10 × 2.1-mm ID, 0.2 μm). The column oven and autosampler cooling tray temperatures were set at 50 °C and 5 °C, respectively. An injection volume of 1.5 μL was used for all injections. Elution was performed using a 20‐min binary gradient at 400 μL/min: 15–90% B in 7 min, 90–100% B in 5.8 min, 100% B for 5.2 min, back to 15% B in 0.1 min, and 1.9-min equilibration by means of 10 mM ammonium acetate in water (eluent A) and 1 mM ammonium acetate in MeOH (eluent B). Full‐scan HRAM‐MS was performed over the range 80–800 Da (automatic gain control (AGC) target 3E6, maximum inject time 100 ms) at a resolution of 70,000 (full width at half maximum (FWHM)). For HCD first‐order fragmentation (Top3; loop count, 3; dynamic exclusion, 10 s), a data‐dependent MS2 Top3 of each scan at a resolution of 17,500 (FWHM) was used with applied stepped NCEs of 25, 50, and 75 eV (AGC Target 1E5, isolation window 1 Da). Vaporizer heater temperature, capillary temperature, spray voltage, sheath gas, and auxiliary gas were set at 350 °C, 380 °C, + 3.00 kV, 60 arbitrary units, and 20 arbitrary units, respectively.

#### RP-HESI(-) method:

The chromatographic method and mass spectrometry settings were identical to those applied for the RP-HESI( +) method, except for the eluent system (eluent A: 1 mM ammonium fluoride in water; eluent B: MeOH) and the spray voltage (− 3.00 kV).

#### RP-APCI(+) method:

The chromatographic method and mass spectrometry settings were identical to those applied for the RP-HESI( +) method. For positive APCI ionization, vaporizer heater temperature, capillary temperature, discharge current, sheath gas, and auxiliary gas were set at 450 °C, 380 °C, 5.0 µA, 50 arbitrary units, and 5 arbitrary units, respectively.

#### HILIC-HESI(+) method:

LC separation was performed using an Accucore™ HILIC column (150 × 2.1 mm ID, 2.6 μm; Thermo Fisher Scientific) preceded by a HILIC Defender guard filter cartridge (10 × 2.1 mm ID, 2.6 μm). The column oven and autosampler cooling tray temperatures were set at 50 °C and 5 °C, respectively. An injection volume of 1.5 μL was used for all injections. Elution was performed using a 15‐min binary gradient at 500 μL/min: 98–75% B in 7 min, back to 98% B in 1 min, and 7-min equilibration by means of 10 mM ammonium acetate in water (eluent A) and 10 mM ammonium acetate in acetonitrile (eluent B). Mass spectrometry and ion source settings were identical to those applied for the RP-HESI(+) method.

To complement LC-HRAM-MS measurements derived from the Q Exactive platform with collision cross section (CCS) measurements for improving the confidence in the compound identifications, the samples were analyzed in addition on a trapped ion mobility spectrometry quadrupole time-of-flight mass spectrometer (Bruker timsTOF Pro™ MS; Bruker Daltonics, Billerica, MA, USA) in full scan/Parallel Accumulation–Serial Fragmentation (PASEF) mode. The Bruker timsTOF Pro™ MS was equipped with a LC System comprising a Bruker Elute HT Binary Pump, Bruker CTC PAL3 autosampler, and a Bruker Elute column oven (Bruker Daltonics). For all CCS methods, the mass acquisition range was 20–1000 Da with funnel 1 RF of 150 Vpp, collision cell In of ± 140 V for positive and negative methods respectively, quadrupole ion energy 5 eV, Quadrupole low mass 150 Da, and collision cell energy 2 eV. TIMS-PASEF parameters were set to inverse reduced mobility (1/k0) acquisition range 0.45–1.10 V∙s/cm^2^, ion charge control 5E6, ramp time 100 ms, enabled TIMS stepping 1 with collision energy 20 eV, collision RF 700 Vpp, transfer time 70 µs, pre pulse storage time 5 µs and enabled TIMS stepping 2 with collision energy 50 eV, collision RF 200 Vpp, transfer time 20 µs, pre pulse storage time 5 µs. The number of PASEF MS/MS scans per precursor was 2. Chromatographic methods were identical to those applied on the Q Exactive platform. Drying gas temperature, drying gas flow, nebulizer gas pressure, capillary voltage, and end plate offset were set at 220 °C, 10 L/min, 2.2 bar, 4500 V, and 500 V, respectively, for the RP-ESI(+) and HILIC-ESI(+) methods, and at 220 °C, 10 L/min, 2.2 bar, − 3500 V, and 500 V, respectively, for the RP-ESI(-) method. For the RP-APCI(+) method, drying gas temperature, drying gas flow, nebulizer gas pressure, capillary voltage, end plate offset, APCI vaporizer temperature, and corona discharge current were set at 220 °C, 4 L/min, 2.5 bar, 4000 V, 500 V, 450 °C, 5000 nA, respectively.

In addition to the untargeted analysis, the per-item yields of 2-furanmethanol, furfural, 3-MCPD, glycidol, and nicotine were analyzed for both test items at Labstat International Inc. (Kitchener, ON, Canada), an ISO 17025-accredited laboratory under contract to PMI. For these analyses, test items were conditioned under the environmental conditions specified in ISO 3402 [17], and the HCI puffing regime was applied [18]. Reported values are the averages of four or three independent determinations for THS and CC, respectively. Further descriptions on the applied methods and their respective accreditation status are available in the Electronic Supplementary Material (Table S2).

### Data processing

Raw data processing was performed using ChromaTOF (LECO Corporation, Saint Joseph, MI, USA) and Progenesis QI™ (version 3.0, Nonlinear Dynamics, Waters Corp., Milford, MA, USA) software for GC × GC-TOFMS and LC-HRAM-MS, respectively. This processing comprised chromatogram alignment and the extraction of chromatographic peak information (retention times and peak areas), as well as of mass spectral information (deconvoluted electron impact (EI) spectra and MS^2^ fragmentation spectra for GC × GC-TOFMS and LC-HRAM-MS, respectively), followed by a selection of compounds with significantly increased abundances in THS samples vs. CC samples by applying a two-tailed two-sample *t*-test (heteroscedastic, significance threshold *P* ≤ 0.05) on the replicate values. Compounds for which the average concentration in the blank samples was higher than 50% of the higher of the averages of THS or CC were excluded. From the LC-HRAM-MS data sets, also those analytical features with no associated MS^2^ spectrum or with a chromatographic peak width below a set limit (0.1 min) were excluded. When multiple LC-HRAM-MS features with identical retention time were detected, the most intense was considered for further evaluation and the rest were discarded to avoid multiple features from the same analyte due to adducts or in-source fragments.

For the statistical evaluation of the THS/CC yield ratio distribution, an analytical feature was defined as a GC × GC 2D peak or LC-HRAM-MS chromatographic peak (defined by m/z and retention time) detected in at least one THS or CC replicate with any of the instrumental methods. The same filtering criteria as mentioned above were applied, except the *t*-test. In addition, we excluded GC × GC or LC features not detected in either THS or CC at the same time with non-significant concentration values for the other test item (i.e., a 95% confidence interval of the concentration mean including the zero value).

Semi-quantitative compound concentrations in the injected sample solutions were estimated based on a comparison of analyte peak areas to peak areas of method-specific (and for GC × GC, also compound class-specific) internal standards of known concentrations (see Electronic Supplementary Material, Tables S6, S7, S8, and S9). For GC × GC–MS data, area calculations were performed with total ions (“Nonpolar” method) or apexing ions (“Polar” and “Volatile” methods) of the deconvoluted mass spectrum of each peak; for internal standards, predefined masses were used. For LC–MS-detected compounds, the extracted ion chromatogram (EIC) of a single mass including its isotope satellites was used. In the present study, only compounds with significantly higher abundance in THS than in CC and at the same time with a per-item yield of at least 37.5 ng in THS are identified and reported, as compounds with a lower yield are of lesser toxicological relevance.^1^ Likewise, we excluded compounds with a THS/CC yield ratio below 1.25, as these have a high probability of appearing significantly different due to the analytical variability, as well as the major aerosol constituents (nicotine, glycerin, propylene glycol, and triacetin).

### Compound identification

Identification of GC × GC-detected compounds was performed by comparison of experimental mass spectral and retention data to an in-house database of reference compounds and to published databases (see Electronic Supplementary Material, Table S3) applying an advanced computer-assisted structure identification (CASI) tool [20, 21]. An “identification confidence” was assigned to each identified compound, with “confirmed” denoting that a close match of EI spectrum as well as 1st and 2nd dimension retention times was observed with those of an authentic reference standard analyzed using the same method. For non-confirmed compounds, “medium” and “high” indicate identifications based on increasing CASI score (700 ≤ CASI score < 795 and CASI score ≥ 795, respectively), which is derived from the mass spectral match factor, retention time similarity, and quantitative structure property relationship models [21].

The identification of LC-HRAM-MS-detected compounds was performed employing searches against an in-house database of experimental retention times and MS^2^ fragmentation data, several public databases of MS^2^ fragmentation data (see Electronic Supplementary Material, Table S4), and in silico predicted fragments of chemical compounds registered in multiple public chemical compound repositories (see Electronic Supplementary Material, Table S5). All putative hits were scored using Progenesis QI™ algorithms, calculating an identification score (IS) considering mass accuracy, isotope similarity, fragmentation score (FS), and retention time. Combined IS and FS threshold criteria were applied to assign confidence levels for compound identification, and proposed compound hits were designated as being identified with “high” (IS > 50 or (50 > IS > 45 and FS > 45)), “medium” (IS < 45 and 20 < FS < 45), or “low” (IS < 45 and FS < 20) confidence [19]. Chemical compounds with both retention times and fragmentation spectra in close agreement with those of purchased reference standards analyzed with the same analytical method were designated as having “confirmed” identification status. For each compound, the likelihood of the proposed molecular structure being a correct identification was further assessed using ion mobility measurements. Each compound was assigned to its associated signal in the Bruker data set based on retention time, accurate mass, and MS^2^ spectrum. Subsequently, the experimental CCS value was extracted from the Bruker data and compared with an in-house CCS reference database. If no CCS reference value based on commercial standards was available, the CCS prediction function of the AllCCS webserver [22, 23] was used. A CCS deviation < 1% to the reference database or < 3% to a predicted CCS reference value further strengthened the confidence in the proposed molecular structure, whereas with higher deviations the next best proposed molecular structures were further investigated.

Following an expert assessment of all proposed identifications, results from the GC × GC-TOFMS and LC-HRAM-MS analyses were combined into one list of differential compounds and their semi-quantitative concentrations per test item. For compounds detected on both instrumental platforms, averaged concentrations (arithmetic mean) are reported. To account for the non-chiroselective nature of the analytical methods, Chemical Abstracts Service (CAS) registry numbers are in general given for the “flat” (non-stereospecific) representations of the chemical structures, except for cases in which CAS numbers only for specific stereoisomers are available, or if the specific stereoisomer could be identified. “Yield ratio” values for each reported compound were calculated as the ratio of per-item yields from the test item (THS) and the comparator item (CC); compounds detected in THS aerosol but not in CC smoke were marked as “unique.”

## Results

### THS/CC yield ratio distributions

We focused on the characterization of aerosol constituents with significantly higher abundances in THS aerosol than in CC smoke. A detailed annotation of aerosol constituents with significantly lower abundances would require a considerably higher effort, mainly for the expert evaluation of the additional compound identification proposals. Nevertheless, a straightforward overall assessment of the degree of difference between test items is possible by evaluating the distribution of THS/CC yield ratios for all analytical features. The yield ratio in this context was defined as the mean per-item yield from the THS replicates divided by the mean per-CC yield from the CC replicates; compounds detected only in either THS or CC are categorized as “unique.” This evaluation was performed on the combined features of the GC × GC-TOFMS and LC-HRAM-MS data sets; however, it should be noted that these analytical features are not fully equivalent to verified aerosol constituents because the data set at this point of the processing workflow has not been subjected to the manual quality check required for eliminating analytical artifacts and duplicate features arising from the same compound detected with different instrument methods.

A classification of all analytical features by their increase or decrease in THS vs. CC, by the statistical significance (*t*-test, *P* ≤ 0.05), and also by their uniqueness in one of the test items (i.e., absence in the samples of the other test item) shows that 92.6% of features are either unique for CC smoke or significantly higher in CC smoke than in THS aerosol, 3.3% are significantly increased in THS aerosol, and only 0.2% are unique for THS aerosol (Table 2). It is noteworthy that in the LC–MS data set, no features unique to one of the samples were detected, while 37% of all GC–MS features fall in this category. This is mainly the result of the “gap-filling” algorithm applied by the LC–MS data processing software, which largely avoids zero values and is not available as an option in the GC–MS software used in this study.

**Table 2:** Number of analytical features by direction of increase and statistical significance (*P* ≤ 0.05)

	LC–MS features	GC–MS features	Total features	Fraction of total features [%]
Unique in CC	0	584	584	15.8
CC > THS (sign.)	2020	825	2845	76.8
Difference non-sign	15	129	144	3.9
THS > CC (sign.)	69	55	124	3.3
Unique in THS	0	7	7	0.2

**Fig. 2:**
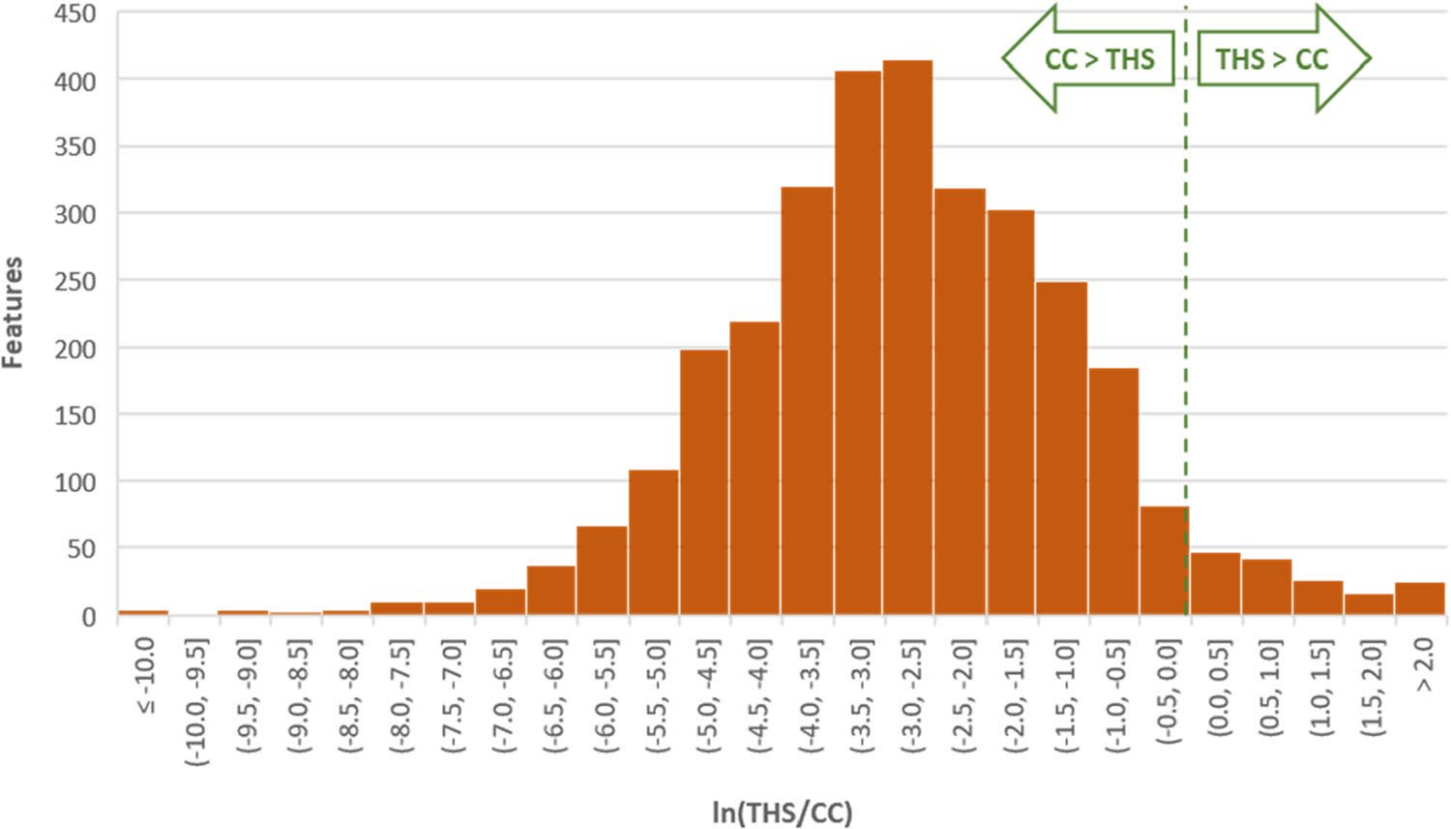
Distribution of yield ratios of all analytical features detected in both THS and CC (bar height indicates the number of features within a given logarithmic yield ratio interval)

For a visualization of the yield ratio distribution, the values were also charted on a histogram depicting the number of features within given intervals along the yield ratio axis (Fig. 2). To obtain maximal coverage of the yield ratios observed in the data set, their natural logarithm is used in the histogram. Consequently, features with identical abundance in the test item and comparator appear at ln(1) = 0. Features unique to either the test item or comparator are not included in the histogram, as the yield ratio for unique features either is undefined (division by zero) or has a value of zero that cannot be represented on a logarithmic scale. This graphical representation demonstrates that the yield ratios for the comparison of THS vs. CC follow an approximately log-normal distribution, which is strongly shifted towards higher yields in CC smoke. The median of the yield ratios for non-unique features lies at 0.06, which is equivalent to a 94% lower yield from THS than from CC. When considering the full set of detected features, including those unique for either THS or CC, the median is at 0.04 (96% reduction).

It is important to note that the observed variance of yield ratios is in part due to the effect of sample preparation and analytical variability. Therefore, even if identical test items were compared with each other, there would be a certain spread of ratios (logically centered around one). To minimize the number of compounds falsely reported as significantly different due to these sources of variability, we applied a yield ratio cutoff of 1.25 for the detailed annotation of compounds with higher abundance in THS aerosol than CC smoke.

### Compounds with higher yields from THS than from CC

For the small fraction of analytical features with higher abundance in THS than in CC or uniquely present in THS, a detailed evaluation was performed that involved removal of duplicates and artifacts followed by a structural identification based on mass spectral data, retention times, and—for features detected by LC–MS—CCS information. This evaluation was limited to features whose concentrations (estimated based on peak area comparisons against internal standards of known concentration) exceeded a threshold of 37.5 ng/item for THS and had a statistically significant (*P* ≤ 0.05) increase of at least 25% in THS aerosol when compared to CC smoke.

Applying these criteria, a total of 31 constituents of the THS aerosol were found to be higher in abundance than in CC smoke (Table 3), with per-item yields ranging from 0.03 to 81.9 μg. Out of these 31 constituents, the chemical structure was confirmed for 29 compounds by comparison of chromatographic retention times and mass spectra to those of authentic standards. A tentative identification is provided for one compound, while one remains unidentified. The unidentified aerosol constituent (compound 16 in Table 3; THS yield, 2229 ng/item) was detected using the Nonpolar and Polar GC × GC-TOFMS methods, and the accurate mass of the presumed molecular ion suggests the sum formula C_5_H_6_O_2_.

**Table 3:** Aerosol constituents with higher per-item yields from THS than CC

No	Proposed compound name	CAS	Identification confidence	Mean yield THS (ng/item)	Mean yield CC (ng/item)	Yield ratio	Analytical platform	Putative glycerol reaction product	Detected in MRTPA^*a*^
1	1-Hydroxy-2-propanone/1,2-Propenediol	116-09-6/7333-03-1	Confirmed	81,906	30,483	2.69	GC	✓	✓
2	1-/2-Monoacetin	106-61-6/100-78-7	Confirmed	52,472	18,708	2.80	LC & GC	✓	✓
3	3-(2-Hydroxymethoxy)-propane-1,2-diol	10548-24-0	Confirmed	41,444	27,241	1.52	GC	✓	
4	2-Furanmethanol	98-00-0	Confirmed	24,500	4750	5.16	Targeted^*b*^		✓
5	4-Cyclopentene-1,3-dione	930-60-9	Confirmed	9491	1466	6.47	GC		✓
6	5-Methylfurfural	620-02-0	Confirmed	8515	1718	4.96	LC & GC		✓
7	1,2-Propanediol, 3-chloro-	96-24-2	Confirmed	7293	2258	3.23	Targeted^*b*^	✓	✓
8	Diacetin	25395-31-7	Confirmed	7226	2406	3.00	LC & GC	✓	✓
9	2-Propanone, 1-(acetyloxy)-	592-20-1	Confirmed	6485	1736	3.74	GC	✓	✓
10	Glycidol	556-52-5	Confirmed	3580	1890	1.89	Targeted^*b*^	✓	✓
11	Benzaldehyde	100-52-7	Confirmed	3260	1956	1.67	GC		
12	Acetoin	513-86-0	Confirmed	2930	1145	2.56	GC		
13	trans-5-Isopropenyl-2-methyl-2-vinyltetrahydrofuran	54750-70-8	Confirmed	2835	627	4.52	GC		✓^*c*^
14	Phenylacetaldehyde	122-78-1	Confirmed	2774	582	4.77	GC		✓
15	2-Acetylfuran	1192-62-7	Confirmed	2451	984	2.49	GC		
16	Not identified (proposed sum formula: C_5_H_6_O_2_)		Not identified	2229	332	6.71	GC		✓^*d*^
17	Menthol	1490-04-6	Confirmed	1891	742	2.55	GC		✓
18	cis-4-Hydroxymethyl-2-methyl-1,3-dioxolane	3674-21-3	Confirmed	1651	44	37.12	GC	✓	✓^*e*^
19	2,3-Dihydroxypropyl propionate	624-47-5	Confirmed	1631	568	2.87	GC	✓	
20	Butyrolactone	96-48-0	Confirmed	1626	411	3.96	GC		✓
21	Glycidyl acetate	6387-89-9	Confirmed	1280	414	3.09	LC	✓	✓
22	2(5H)-Furanone	497-23-4	Confirmed	1166	394	2.96	GC		✓
23	Dimethyl trisulfide	3658-80-8	Confirmed	823	543	1.52	GC		
24	3(2H)-Furanone, dihydro-2-methyl-	3188-00-9	Confirmed	820	356	2.31	GC		✓
25	1,3-Propanediol, 2-chloro-	497-04-1	Confirmed	651	291	2.23	GC	✓	
26	Methyl 2-furoate	611-13-2	Confirmed	635	87	7.32	GC		✓
27	2-Methylvaleric acid	97-61-0	Confirmed	537	66	8.09	LC		
28	Butylated hydroxytoluene (BHT)	128-37-0	Confirmed	477	0	Unique	GC		✓
29	2(3H)-Furanone, 5-methyl-	591-12-8	Confirmed	429	247	1.74	GC		
30	3-(Furfuryloxy)-1,2-propanediol	20390-21-0	Low	421	288	1.46	LC	✓	
31	3-Buten-2-ol, 2-methyl-	115-18-4	Confirmed	321	186	1.72	GC		

^*a*^Reported in NTDS studies performed for THS 2.2 MRTPA as increased vs. 3R4F for at least one of the three tested variants

^*b*^Analyzed using validated targeted methods

^*c*^Previously reported as the specific stereoisomer anhydrolinalool oxide

^*d*^Previously reported as 2-methylcyclobutane-1,3-dione (incorrect identification)

^*e*^Previously reported as trans isomer

After reintegration of the seven GC features uniquely found in THS with a lower signal-to-noise threshold, traces of six of these features were found to be present also in the CC samples. The only compound confirmed as unique to THS (i.e., not detected in CC) was butylated hydroxytoluene (BHT; THS yield, 477 ng/item). BHT was most likely not released from the heated tobacco substrate but transferred to the aerosol from a paper adhesive used in the THS item, in which it was present as an antioxidant ingredient. It should be noted that BHT was detected in aerosol of THS 2.2 variants (132, 154, and 149 ng/item) and in smoke of the 3R4F reference cigarette (7 ng/item) in a previous NTDS study [5].

For the non-unique constituents, the yield ratios (i.e., the per-stick yields) from THS divided by those from CC are provided in Table 3. With a yield ratio above 37, cis-4-hydroxymethyl-2-methyl-1,3-dioxolane displayed the highest relative difference between THS and CC. For five further compounds, yield ratios between 5.0 and 10 were observed, 17 compounds exhibit yield ratios between 2.0 and 5.0, and the remaining seven constituents showed only moderate differences between THS and CC, with yield ratios below 2.0.

Four toxicologically relevant compounds highlighted previously as being present in THS aerosols in higher concentrations than in cigarette smoke were quantified in the present study using validated methods. Three of these (glycidol, 3-MCPD, and 2-furanmethanol) were again confirmed as increased, and the per-item yields listed in Table 3 are those obtained with these methods. For furfural, the yield difference between THS and CC was not statistically significant (THS, 25.0 ± 3.0 µg/item; CC, 23.8 ± 0.7 µg/item; *P* = 0.48). Likewise, nicotine was quantified with a validated method at 1.29 ± 0.04 and 2.55 ± 0.12 mg/item for THS and CC, respectively.

The traces of menthol detected at low concentrations in both the THS (1891 ng/item) and CC (742 ng/item) likely originated from contamination during manufacturing of these items in a facility that also produces mentholated products; menthol vapors in the production environment cannot be excluded.

## Discussion

The tobacco substrate in THS 2.2 is heated to a maximum temperature of 350 °C [2], which is significantly lower than the temperatures reached in the burning tobacco cone of a cigarette, which can reach 900 °C during a puff [24]. Due to this temperature difference, cigarette smoke contains a much more complex mixture of chemical compounds resulting from combustion and high-temperature pyrolysis. However, as the temperature of tobacco in a burning cigarette decreases with longer distance from the tip, there are temperature zones where the same low-temperature pyrolysis reactions and distillation processes that occur in THS contribute to the chemical makeup of the smoke. Thus, while there may be quantitative differences in the levels of certain constituents between THS aerosol and cigarette smoke, it is reasonable to assume that THS aerosol contains only compounds that are also present in cigarette smoke. It is important to note that the non-tobacco ingredients used in THS tobacco substrate can be disregarded as potential sources of pyrolysis products absent in cigarette smoke: polysaccharides identical or very similar to cellulose and guar gum (used in THS substrate to ensure optimal mechanical properties) are also natural tobacco constituents, and glycerol (which serves as an aerosol former in THS substrate) is applied in most commercial cigarettes as a moisture control agent.

With the comprehensive set of methods applied in the present study, no compounds were detected that originated from heating a tobacco substrate in THS 2.2 that were absent in smoke from a cigarette manufactured from the same tobacco. BHT—the sole compound uniquely detected in THS aerosol—is in all likelihood not released from the tobacco substrate but leaches into the THS aerosol from a paper adhesive. The detection of BHT in this and a previous study [5] demonstrates that non-targeted screening methods have the power to detect and identify low concentration leachables in complex matrices; however, standard protocols for extractable identification in manufacturing materials in combination with targeted leachable analyses in the aerosol matrix are arguably a more efficient strategy for achieving the same goal.

Since the tobacco blend used in the present study contains all major tobacco types (Burley, flue-cured, Oriental, and air-cured), the results of this study are expected to be also representative for THS variants with other tobacco blends. This generalization is somewhat corroborated by a previously published study investigating the effect of varying THS blends on the aerosol yields of a broad set of chemically diverse HPHCs, which concluded that for most of these HPHCs “the tobacco blend composition had only a minimal impact on the yields (…) in the resulting aerosols.” [14]. Assuming that the mechanisms responsible for the chemical composition of an HTP aerosol are independent of the specific heating device but rather a function of the heating temperature, the results presented here also support the generalized notion that heating tobacco to temperatures equal to or lower than those in THS 2.2 is unlikely to give rise to aerosol constituents that are not also present in smoke created by burning the same tobacco.

The levels of constituents present in both THS aerosol and cigarette smoke can vary between the two matrices due to different formation, release, and degradation kinetics. A statistical evaluation of the totality of analytical features detected in both THS and CC samples showed that the majority of these common aerosol/smoke constituents are present at markedly lower levels in THS aerosol. The observed median reduction of 94% in THS vs. CC is in good agreement with results of a previous study, which found a > 90% reduction for the majority of analyzed HPHCs in THS 2.2 aerosol compared to smoke of the 3R4F reference cigarette [2].

The main factors determining the per-item yield differences between the THS and CC are plausibly the temperature–time profile the tobacco is exposed to and the amount of tobacco available as a reservoir for aerosol constituents and their precursors, but the nature and concentrations of non-tobacco ingredients can also play an important role. In this study, one difference between THS and the CC was the level of glycerol in the tobacco substrate. The THS tobacco substrate contained 17.7% (w/w dry weight basis) of glycerol, which was thus the second most abundant ingredient in the reconstituted tobacco sheet, where it serves as an aerosol former and is essential for the manufacturability of the material. In contrast, the tobacco cut filler in the CC contained 1.7% of added glycerol—a level representative for the glycerol content of marketed cigarettes. Considering this difference in glycerol content, it was not surprising that a notable fraction (12 out of 31; 39%) of the compounds with higher levels in THS aerosol were putative glycerol reaction products: esters (monoacetins, diacetin, 2,3-dihydroxypropyl propionate), dehydration products (1-hydroxy-2-propanone, glycidol, and—through dehydration of monoacetins—glycidyl acetate and 1-acetyloxy-2-propanone), acetals, and hemiacetals formed by reactions of glycerol with small aldehydes (glycerol + formaldehyde → hydroxymethoxy-1,2-propanediol; glycerol + acetaldehyde → cis-4-hydroxymethyl-2-methyl-1,3-dioxolane), and products of a nucleophilic substitution (3-chloro-1,2-propanediol and 2-chloro-1,3-propanediol). The glycerol-formaldehyde hemiacetal should not be interpreted as a source of hidden formaldehyde, since the derivatization reagent commonly used for the quantitation of aldehydes in tobacco smoke or aerosols is also able to react with the glycerol hemiacetal of formaldehyde [25], and the same can be reasonably assumed for acetaldehyde and its glycerol acetal. Therefore, also the aldehyde levels previously reported [2] for THS 2.2 aerosol represent the sums of the respective free and glycerol-bound analytes.

Nine out of the 31 compounds with higher yields from THS than the CC contain a furan moiety. 2-Furanmethanol and 5-methylfurfural are known products of the thermal decomposition of sugars [26], and their presence in THS aerosol is probably due to the naturally occurring glucose, fructose, and polysaccharides in tobacco. The higher levels of some furans in THS aerosol—despite the same tobacco being used in both test items—can be seen as a consequence of the temperature conditions in THS being more favorable for their formation and intact transfer to the aerosol.

Within the context of the MRTPA for THS 2.2, similar non-targeted comparisons between THS aerosol and cigarette smoke were previously conducted [5]. These analyses involved three flavored THS variants and the 3R4F research cigarette, which has a different tobacco blend structure and is unflavored. Compared to the present study, the results showed a higher number of compounds detected as increased in THS aerosol vs. the research cigarette, even when retrospectively applying the same per-stick-yield and yield ratio thresholds as in the present study (present study, 31 compounds; THS non-mentholated > 3R4F, 47 compounds; THS low menthol > 3R4F, 49 compounds; THS high menthol > 3R4F, 57 compounds). The smaller number of increased compounds detected in the current study is likely due to the exclusion of potential sources of differences not inherent to the compared product types (i.e., flavors and tobacco blend). As expected, there was a significant overlap between the set of compounds identified as higher in THS than in CC in the present study and the compounds reported for the MRTPA, with 20 out of the 31 aerosol constituents with higher yields from THS than from CC also detected in at least one of the three product variants analyzed for the MRTPA as increased vs. 3R4F smoke (Table 3). The majority of the remaining 11 compounds were detected in the previous NTDS study but were either higher in 3R4F smoke or the increase in the THS aerosols was not significant.

These findings demonstrate that untargeted comparisons of aerosol or smoke from tobacco products can be carried out in a more controlled and unbiased manner by excluding sources of differences that are not fixed properties of the product class under investigation. This approach can help researchers focus on differences related to the heating technology and product design, which are typically fewer than those found in less controlled comparisons.

The outcomes of this study must be considered in the context of its technical limitations. While the applied LC- and GC–MS methods effectively cover a wide range of chemical space, certain compound classes remain beyond their scope (e.g., metals, gases like NO or CO, and highly reactive species including radicals). Moreover, despite the ability of our methods to detect analytes in the ng/item range, it is crucial to acknowledge that some toxicologically relevant compounds, such as tobacco-specific nitrosamines, might fall below this sensitivity threshold. However, quantitative levels of several of the aerosol constituents in THS 2.2 aerosol not captured by the untargeted method portfolio were previously published [2]. Implementing new analytical technologies and data processing tools can help further optimize the untargeted analysis of tobacco products. For example, improving compound coverage in LC–MS/MS could be pursued through approaches like data-independent acquisition (DIA), which would eliminate biases due to precursor ion selection and cycle times but might make it more challenging to extract the high-quality information (i.e., clean fragment spectra linked to information about the precursor) needed for compound identification through searches in publicly available databases. This study provides evidence that heating tobacco produces only a subset of the chemicals found in cigarette smoke and that most of the constituents present in both matrices are substantially less abundant in THS aerosol. Additionally, it contributes to a clearer understanding of the array of compounds users of HTPs may be exposed to in potentially higher concentrations than cigarette smokers due to differences in the mechanisms of aerosol or smoke generation. Building on these findings, further experiments may be designed to characterize formation mechanisms and the influence of operating temperature and other product design features on compound yields. Finally, it is important to note that the comprehensive characterization of aerosol chemistry constitutes just the first step of the toxicological risk assessment of HTPs, which must be followed by an evaluation of the toxicological hazards arising from the identified aerosol constituents and complemented by the toxicological evaluation of single ingredients and in vitro bioassay results of the HTP aerosol [27].

### Supplementary Information

Below is the link to the electronic supplementary material.

Supplementary file1 (PDF 1567 KB)

## Footnotes

1 The limit is based on a toxicological threshold of concern (TTC) approach, considering a TTC of 1.5 µg per person per day as the lowest safety concern threshold for the potential presence of mutagenic compounds, similar to the guidance of ICH M7 (R2) for the identification of mutagenic impurities in pharmaceuticals. Assuming an exaggerated daily product use of 40 sticks per day, this translates into a threshold of 37.5 ng/stick.
