# Supplementary material for: Non-targeted analytical comparison of a heated tobacco product aerosol against mainstream cigarette smoke: does heating tobacco produce an inherently different set of aerosol constituents?
Source: Anal Bioanal Chem. 2024 Jan 13;416(6):1349–61. doi: 10.1007/s00216-024-05126-x (PMC10861380; doi:10.1007/s00216-024-05126-x)
Supplement: Supplementary file 1 — Supplementary file1 (PDF 1567 KB) [file 216_2024_5126_MOESM1_ESM.pdf]

ELECTRONIC SUPPLEMENTARY MATERIAL

for

Non-Targeted Analytical Comparison of a Heated Tobacco Product Aerosol Against Mainstream Cigarette Smoke: Does Heating Tobacco Produce an Inherently Different Set of Aerosol Constituents?

Gerhard Lang, Carlos Henao, Martin Almstetter, Daniel Arndt, Catherine Goujon, Serge Maeder

PMI R&D, Philip Morris Products S.A., Quai Jeanrenaud 5, CH-2000, Neuchâtel, Switzerland

Corresponding Author

Gerhard Lang: gerhard.lang@pmi.com

Table S1 Chemicals, internals standards (ISTDs) and retention index markers (RIMs) used in GC×GC-TOFMS and LC-HRAM-MS analyses

	**Supplier**
GC×GC-TOFMS chemicals:	
Acetone, dichloromethane, N,N-dimethylformamide, hexachlorobenzene, sodium sulfate, and water	Merck KGaA (Darmstadt, Germany)
GC×GC-TOFMS Nonpolar method ISTDs:	
2,3,5-Trimethylpyrazine-d_{10}, 2-nonanone-1,1,1,3,3-d_5, 4-tert-butylbenzoic-d_{13} acid, 5-hydroxy-2-methyl-d_3-pyridine-3,4,6-d_3, cholesterol-2,2,3,4,4,6-d_6, decanoic-d_{19} acid, dibenzofuran-d_8, ethyl dodecanoate-d_{23}, isophorone-d_8 (2,4,4,6,6-d_5; 3-methyl-d_3), isoquinoline-d_7, myosmine-d_4 (pyridine-d_4), N,N-dimethyl-d_6-acetamide, N-methylpyrrole-d_4 (ring-d_4), nonanal-d_{18}, n-pentadecane-d_{32}, phenol-d_6, tert-butylbenzene-d_{14}, and n-tetradecyl-d_{29} alcohol	CDN Isotopes Inc. (Pointe-Claire, Quebec, Canada)
GC×GC-TOFMS Nonpolar method RIMs:	
n-Heptane-d_{16}, n-octane-d_{18}, n-nonane-d_{20}, n-decane-d_{22}, n-dodecane-d_{26}, n-pentadecane-d_{32}, n-eicosane-d_{42}, n-pentacosane-d_{52}, n-triacontane-d_{62}, n-dotriacontane-d_{66}, n-tetratriacontane-d_{70}, n-hexatriacontane-d_{74}, and n-octatriacontane-d_{78}	CDN Isotopes Inc.
GC×GC-TOFMS Polar method ISTDs:	
Furfural-d_4, 4-hydroxy-4-methyl-2-pentanone-d_{12}, 5-hydroxy-2-methyl-d_3-pyridine-3,4,6-d_3, N-methylnicotinamide-2,4,5,6-d_4, (±)-2-methylbutyric-d_9 acid, (±)-2-methyl-2,4-pentane-d_{12}-diol, pentanenitrile-d_9, phenol-d_6, N-isopropyl-d_7-acrylamide, and pyridine-d_5	CDN Isotopes Inc.
GC×GC-TOFMS Polar method RIMs:	
Methylhexanoate and methylarachidate	Merck KGaA
Methyldecanoate-d_{19}, methyl tetradecanoate-d_{27}, and methylhexadecanoate-d_{31}	CDN Isotopes Inc.
GC×GC-TOFMS Volatile method ISTDs:	
Acetone-d_6, benzene-d_6, butyraldehyde-d_8, 1,2-dichloroethane-d_4, dimethyl sulfide-d_6, ethyl acetate-d_8, furan-d_4, n-hexane-d_{14}, methacrylonitrile-d_5, 3-methylhexane-d_{16}, tetrahydrofuran-d_8 were obtained from Merck KGaA. 2-Butanone-1,1,1,3,3-d_5, cyclohexene-d_{10}, cyclopentane-d_{10}, and (±)-1,2-propylene-d_6 oxide	CDN Isotopes Inc.
GC×GC-TOFMS Volatile method RIMs:	
n-Hexane-d_{14}	Merck KGaA
n-Pentane-d_{12} and n-heptane-d_{16}	CDN Isotopes Inc.
LC-HRAM-MS chemicals:	
LC/MS-grade acetonitrile (ACN), formic acid, ammonium fluoride, sodium hydroxide, and ammonium acetate	Sigma-Aldrich (St. Louis, MO, USA)
LC/MS-grade methanol (MeOH), isopropyl alcohol, and water	Honeywell Fluka (Charlotte, NC, USA)
LC-HRAM-MS ISTDs:	
Decanoic-d_{19}-acid, isophorone-d_8 (2,4,4,6,6-d_5; 3-methyl-d_3) and myosmine-d_4 (pyridine-d_4)	CDN Isotopes Inc.

Table S2 Analytical methods applied at Labstat International Inc. for the targeted quantification of selected aerosol constituents

Method Description	Reportable Analytes	Method Number, Accreditation status
2-MCPD and 3-MCPD in Mainstream Heat-not-Burn Emissions by GC-MS (SIM), Full Panel - Linear Smoking	3-chloro-1,2-propanediol (3-MCPD); battery/device ID; item weight; puff count; TPM	TMS-00185,<br>ISO 17025
Furfural and 2-furanmethanol in Mainstream Heat-not-Burn Emissions by GC-MS (SIM), Full Panel - Linear Smoking	2-furanmethanol; furfural; battery/device ID; item weight; puff count; TPM	TMS-00187,<br>ISO 17025
Glycidol in Mainstream Heat-Not-Burn Emissions by GC-MS (SIM) - Linear Smoking	glycidol; battery/device ID; item weight; puff count; TPM	TMS-00115a Appendix I (modified),<br>ISO 17025
2-MCPD and 3-MCPD in Mainstream Tobacco Smoke by GC-MS (SIM), Full Panel – Linear Smoking	3-chloro-1,2-propanediol (3-MCPD); item weight; puff count; TPM	TMS-00185,<br>ISO 17025
Furfural and 2-furanmethanol in Mainstream Tobacco Smoke by GC-MS (SIM), Full Panel - Linear Smoking	2-furanmethanol; furfural; item weight; puff count; TPM	TMS-00187,<br>ISO 17025
Glycidol in Mainstream Tobacco Smoke by GC-MS (SIM), Panel 3 - Linear Smoking	glycidol; item weight; puff count; TPM	TMS-00115a Appendix I (modified),<br>None
Tar, Nicotine and Carbon Monoxide in Mainstream Tobacco Smoke by GC-TCD/FID, Health Canada List - Linear Smoking	FDPM; nicotine; water; carbon monoxide; Item Weight; puff count; TPM	TMS-00115,<br>ISO 17025

Table S3 Commercial mass spectral databases used in the computer-assisted structure identification (CASI) process for compound identification from GC×GC-TOFMS data

Mass Spectral Database	No. of Mass Spectra
Identification of Essential Oil Components by Gas Chromatography/Mass Spectrometry [a]	2205
Mass Spectra of Androgens, Estrogens and other Steroids 2010 [b]	4081
Mass Spectra of Geochemicals, Petrochemicals and Biomarkers [c]	1100
LECO/Fiehn Metabolomics Library [d]	1100
Mass Spectral Library of Drugs, Poisons, Pesticides, Pollutants and Their Metabolites [e]	10,430
FFNSC 3 – Mass Spectra of Flavour & Fragrance Natural & Synthetic Compounds [f]	3462
Wiley Registry of Mass Spectral Data, 12th Edition [g]	817,290
NIST 20 Mass Spectral Library [h]	350,643

[a] Adams, R.P., Identification of Essential Oil Components by Gas Chromatography/Mass Spectrometry, 5th ed.; *Texensis Publishing*: Gruver (TX, USA), 2017. ISBN 978-0-9981557-2-2

[b] Parr, M.K., Opfermann, G., Schänzer, W., Makin, H. L. J., Mass Spectra of Androgens, Estrogens, and other Steroids 2010.; *Wiley-VCH*: Weinheim (Germany), 2010. ISBN 978-3527327270

[c] De Leeuw, J.W., Mass Spectra of Geochemicals, Petrochemicals and Biomarkers.; *Wiley-VCH*: Weinheim (Germany), 2003. ISBN 978-0-471-64798-0

[d] Fiehn, O., LECO/Fiehn Metabolomics Library; *LECO Corporation*: St. Joseph (MI, USA), 2013.

[e] Maurer, H.H., Pfleger, K., Weber, A.W., Mass Spectral Library of Drugs, Poisons, Pesticides, Pollutants and Their Metabolites, 5th ed.; *Wiley-VCH*: Weinheim (Germany), 2017. ISBN 978-3-527-34327-0

[f] Mondello, L., FFNSC 3 - Mass Spectra of Flavour & Fragrance Natural & Synthetic Compounds, 3rd ed.; *Wiley-VCH*: Weinheim (Germany), 2015. ISBN 978-1-119-06984-3

[g] Wiley Registry of Mass Spectral Data, 12th ed.; *Wiley-VCH*: Weinheim (Germany), 2020.

[h] NIST 20 Mass Spectral Library, 12th ed.; *NIST, U.S. National Institute of Standards and Technology*, Gaithersburg (MD, USA), 2020.

Table S1 Mass Spectral Databases used for compound identification from LC-HRAM-MS data

Mass Spectral Database	No. of Mass Spectra
UCSD – PMI's Unique Compound Spectral Database [a]	3,342
NIST 20 MS/MS Mass Spectral Library [b]	30,136
METLIN MS/MS Mass Spectral Library [c]	143,333
MoNA MS/MS Mass Spectral Library [d]	137,441
MS-DIAL Mass Spectral Library [22] [e]	327,763

[a] Martin, E., Monge, A., Duret, JC., Gualandi, F., Peitsch, MC., Pospisil, P. (2012) Building an R&D chemical registration system. *J. Cheminformatics. 4*, 11. https://doi.org/10.1186/1758-2946-4-11

[b] NIST 20 MS/MS. *NIST, U.S. National Institute of Standards and Technology*, Gaithersburg (MD, USA); https://www.nist.gov/programs-projects/nist20-updates-nist-tandem-and-electron-ionization-spectral-libraries

[c] METLIN MS/MS. *METLIN, Scripps Research Institute*, La Jolla (CA, USA); https://metlin.scripps.edu/landing_page.php?pgcontent=mainPage

[d] MoNA MS/MS library. *MassBank of North America (MoNA)*, Fiehnlab, University of California UC Davis, Davis (CA, USA); https://mona.fiehnlab.ucdavis.edu/

[e] MS-DIAL metabolomics mass spectral library. *RIKEN Center for Sustainable Resource Science*, Metabolome Informatics Research Team, Yokohama City, (Kanagawa, Japan); http://prime.psc.riken.jp/compms/msdial/main.html

Table S5 Compound structure databases used for compound identification from LC-HRAM-MS data via *in-silico* fragmentation

**Compound Structure Database**
UCSD – PMI's Unique Compound Spectral Database [a]
HMDB 5.0 [b, c]
COCONUT [d, e]
EPA CompToX [f, g]
ChemIDplus – via ChemSpider search plugin [h]
FDA - via ChemSpider search plugin [i, j, k]
ChEBI - via ChemSpider search plugin [l]
Golm Metabolome Database - via ChemSpider search plugin [m]
KEGG – via ChemSpider search plugin [n]
Nature Chemistry – via ChemSpider search plugin [o]

[a] Martin, E., Monge, A., Duret, JC., Gualandi, F., Peitsch, MC., Pospisil, P. (2012) Building an R&D chemical registration system. *J. Cheminformatics. 4*, 11; https://doi.org/10.1186/1758-2946-4-11

[b] Human Metabolome Database, Edmonton (Canada): *University of Alberta*, Edmonton, HMDB 5.0; https://hmdb.ca/downloads

[c] Wishart, D.S., Guo, AC., Oler, E., Wang, F., Anjum, A., Peters, H., Dizon, R., Sayeeda, Z., Tian, S., Lee, B.L., Berjanskii, M., Mah, R., Yamamoto, M., Jovel, J., Torres-Calzada, C., Hiebert-Giesbrecht, M., Lui, V.W., Varshavi, D., Allen, D., Arndt, D., Khetarpal, N., Sivakumaran, A., Harford, K., Sanford, S., Yee, K., Cao, X., Budinski, Z., Liigand, J., Zhang, L., Zheng, J., Mandal, R., Karu, N., Dambrova, M., Schiöth, H.B., Greiner, R., Gautam, V. (2021) HMDB 5.0: the Human Metabolome Database for 2022. *Nucleic Acids Res.* 50, (D1):D622-D631. https://doi.org/10.1093/nar/gkab1062

[d] COCONUT: the COlleCtion of Open NatUral producTs, *Friedrich-Schiller-University*, Institute for Analytical Chemistry, Cheminformatics and Computational Metabolomics (Jena, Germany): https://zenodo.org/record/3547718#.X7ZZI2hKiHs

[e] Sorokina, M., Steinbeck, C. (2020) Review on natural products databases: where to find data in 2020. *J. Cheminformatics*. 12, 20; https://doi.org/10.1186/s13321-020-00424-9

[f] CompTox, *U.S. Environmental Protection Agency*, Research Triangle Park, Durham (NC, US): https://comptox.epa.gov/dashboard

[g] Williams, A.J., Grulke, C.M., Edwards, J., McEachran, A.D., Mansouri, K., Baker, N.C., Patlewitz, G., Shah, I., Wambaugh, J.F., Judson, R.S., Richard, A.M. (2017) The CompTox Chemistry Dashboard: a community data resource for environmental chemistry. *J. Cheminformatics*. 9, 61.; https://doi.org/10.1186/s13321-017-0247-6

[h] Chemspider Plugin, ChemIDplus data source, *ChemIDplus, SIS, NLM, NIH*, Bethesda (MD, USA); https://chem.nlm.nih.gov/chemidplus/

[i] Chemspider Plugin, FDA data source, *FDA, U.S. Food and Drug Administration*, Silver Spring (MD, USA); https://www.fda.gov/

[j] Chemspider Plugin, FDA UNII - NLM data source*, NLM, U.S. National Library of Medicine*, Bethesda (MD, US); https://fdasis.nlm.nih.gov/srs/jsp/srs/uniiListDownload.jsp

[k] Chemspider Plugin, FDA Structured Product Labeling index data source, *FDA, U.S. Food and Drug Administration*, Silver Spring (MD, USA); https://cactus.nci.nih.gov/download/fda/

[l] Chemspider Plugin, ChEBI data source, Chemical Entities of Biological Interest (ChEBI), *European Bioinformatics Institute*, Hinxton (UK); http://www.ebi.ac.uk/chebi/

[m] Chemspider Plugin, Golm Metabolome Database data source, The Golm Metabolome Database, *Max Planck Institute of Molecular Plant Physiology*, Potsdam (Germany); http://gmd.mpimp-golm.mpg.de/

[n] Chemspider Plugin, KEGG data source, Kyoto Encyclopedia of Genes and Genomes, *Kyoto University Bioinformatics Center*, Kyoto (Japan); http://www.genome.jp/kegg/kegg2.html

[o] Chemspider Plugin, Nature Chemistry data source, *Nature Publishing Group*, (UK); http://www.nature.com/nchem/

Table S2 Assignment of compound classes to specific ISTDs – GC×GC-TOFMS Nonpolar method

ISTD Compound	Concentration in Sample [µg/mL]	Case of Known Compounds	Case of Unknown Compounds
2,3,5-Trimethylpyrazine-d_{10}	1.3	Internal standard for pyrazines	-
2-Nonanone-1,1,1,3,3-d_5	1.3	Internal standard for aliphatic ketones	-
4-Tert-butylbenzoic-d_{13} acid	7.0	Internal standard for cyclic and aromatic acids	-
5-Hydroxy-2-methyl-d_3-pyridine-3,4,6-d_3	5.6	Internal standard for hydroxypyridines	-
Cholesterol-2,2,3,4,4,6-d_6	2.8	Internal standard for polycyclic (sterol) alcohols	-
Decanoic acid-d_{19}	5.5	Internal standard for aliphatic acids	-
Dibenzofuran-d_8	0.8	Internal standard for furans	-
Ethyl dodecanoate-d_{23}	1.4	Internal standard for esters	-
Isophorone-d_8	1.1	Internal standard for cyclic and aromatic carbonyls	Internal standard for compounds with 2nd dimension relative retention time ≤1.5 (in general corresponds to nonpolar to medium polar unknowns)
Isoquinoline-d_7	1.4	Internal standard for pyridines or other N-containing rings	Internal standard for compounds with 2nd dimension retention time >1.8 (in general corresponds to basic unknowns)
Myosmine-2,4,5,6-d_4	2.3	Internal standard for compounds containing 2 or more N-containing rings	-
N,N-Dimethyl-d_6-acetamide	3.1	Internal standard for amides	-
N-Methylpyrrole-d_4	1.5	Internal standard for pyrroles	-
Nonanal-d_{18}	1.3	Internal standard for aliphatic aldehydes	-
Pentadecane-d_{32}	1.5	Internal standard for aliphatic hydrocarbons	-
Phenol-d_6	2.5	Internal standard for phenols and phenyl ethers; dihydroxy-benzenes; bis-, bi- and polyphenols	Internal standard for compounds with 2nd dimension relative retention time >1.5 and ≤1.8 (in general corresponds to medium polar aromatic unknowns)
Tert-butylbenzene-d_{14}	0.7	Internal standard for aromatic hydrocarbons	-
Tetradecyl-d_{29} alcohol	2.8	Internal standard for aliphatic alcohols	-

Table S3 Assignment of compound classes to specific ISTDs – GC×GC-TOFMS Polar method

ISTD Compound	Concentration in Sample [µg/mL]	Case of Known Compounds	Case of Unknown Compounds
Furfural-d_4	0.5	Internal standard for<br>• compounds with at least one ring (without nitrogen) and one carbonyl function<br>• (thio)ethers (without N-containing rings)	Internal standard for compounds with 2DRT >2.5 and ≤4.25 s
4-Hydroxy-4-methyl-2-pentanone-d_{12}	1.3	Internal standard for carbonyls (without a ring)	-
5-Hydroxy-2-methyl-d_3-pyridine-3,4,6-d_3	1.5	Internal standard for compounds with at least one N-containing ring and one oxygen-containing functional group (except amide)	-
N-Methylnicotinamide-2,4,5,6-d_4	1.0	Internal standard for compounds with at least one ring and one amide function	-
2-Methylbutyric-d_9 acid	2.5	Internal standard for acids, thioacids (without N-containing rings)	-
2-Methyl-2,4-pentane-d_{12}-diol	2.0	Internal standard for alcohols (without a ring)	-
Pentanenitrile-d_9	0.8	Internal standard for nitriles	Internal standard for compounds with 2DRT >4.25 s
Phenol-d_6	1.0	Internal standard for compounds with at least one ring and one alcohol (without N-containing rings)	Internal standard for compounds with 2DRT ≤2.5 s
N-iso-Propyl-d_7-acrylamide	0.5	Internal standard for compounds without a ring and at least one amide function	-
Pyridine-d_5	0.5	Internal standard for N-containing rings without oxygen-containing functional groups	-

Table S4 Assignment of compound classes to specific ISTDs – GC×GC-TOFMS Volatile method

ISTD Compound	Concentration in Sample [µg/mL]	Case of Known Compounds	Case of Unknown Compounds
Acetone-d_6	2.5	Internal standard for ketones with RI ≤590	-
Benzene-d_6	2.7	Internal standard for aromatic hydrocarbons	-
2-Butanone-d_5	2.4	Internal standard for ketones with RI >590	-
Butyraldehyde-d_8	2.1	Internal standard for aldehydes	-
Cyclohexene-d_{10}	1.4	Internal standard for unsaturated cyclic hydrocarbons	Internal standard for compounds with 2DrelRT ≤1.173 (in general corresponds to nonpolar unknowns)
Cyclopentane-d_{10}	1.2	Internal standard for saturated cyclic hydrocarbons	-
1,2-Dichloroethane-d_4	2.2	Internal standard for Cl-containing compounds	-
Dimethyl sulfide-d_6	0.6	Internal standard for S-containing compounds	-
Ethyl acetate-d_8	0.9	Internal standard for esters	Internal standard for compounds with 2DrelRT >1.173 and ≤1.788 (in general corresponds to nonpolar to medium polar unknowns)
Furan-d_4	2.7	Internal standard for compounds with at least one ether function and at least one double or aromatic bond	-
Methacrylonitrile-d_5	2.4	Internal standard for N-containing compounds	Internal standard for compounds with 2DrelRT >1.788 (in general corresponds to medium polar to medium polar aromatic or basic unknowns)
3-Methylhexane-d_{16}	1.5	Internal standard for branched hydrocarbons (saturated and unsaturated)	-
Propylene oxide-d_6	2.1	Internal standard for ethers (cyclic and non-cyclic), no double or aromatic bond, RI ≤600	-
Tetrahydrofuran-d_8	1.3	Internal standard for ethers (cyclic and non-cyclic), no double or aromatic bond, RI >600	-
n-Hexane-d_{14}	1.8	Retention-index marker 2, internal standard for linear hydrocarbons (saturated and unsaturated)	Retention-index marker 2

Table S9 Assignment of specific ISTDs – LC -HRAM-MS

ISTD Compound	Concentration in Sample [µg/mL]	Method Assignment
Isophorone-d_8	10.0	RP-HESI positive, RP-APCI-positive
Decanoic-d_{19}-acid	20.0	RP-HESI negative
Myosmine-d_4	5.0	HILIC positive

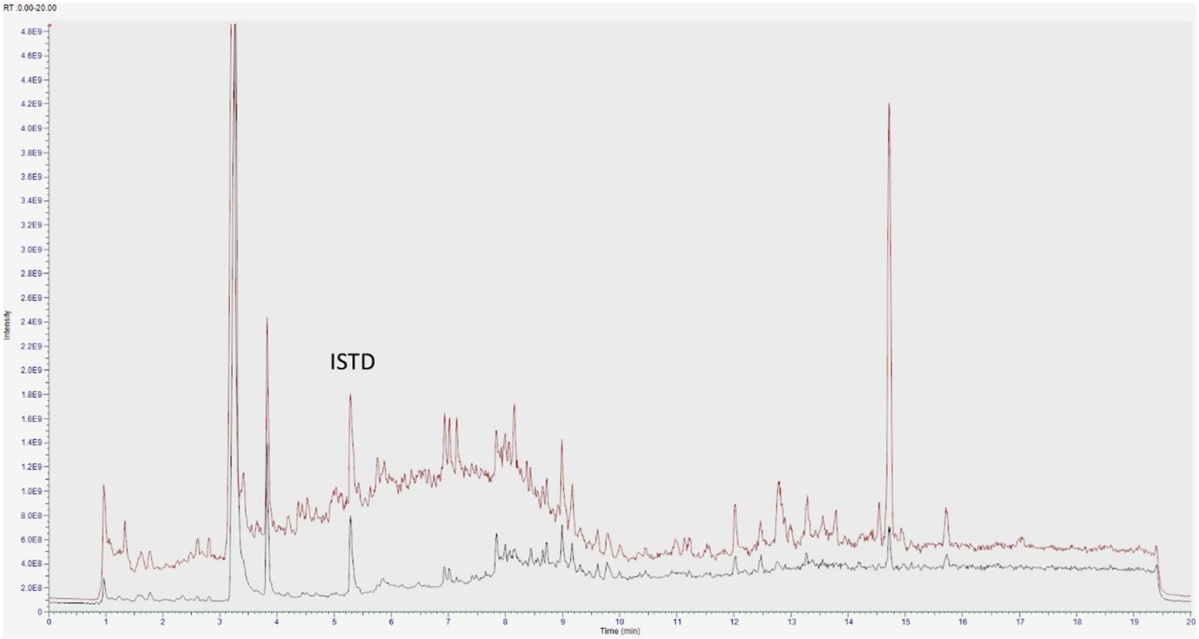

Fig. S1 LC-HRAM-MS total ion chromatograms of THS aerosol (black trace) and CC smoke (red trace) acquired with the RP-HESI(+) method; "ISTD" denotes the peak of the internal standard.

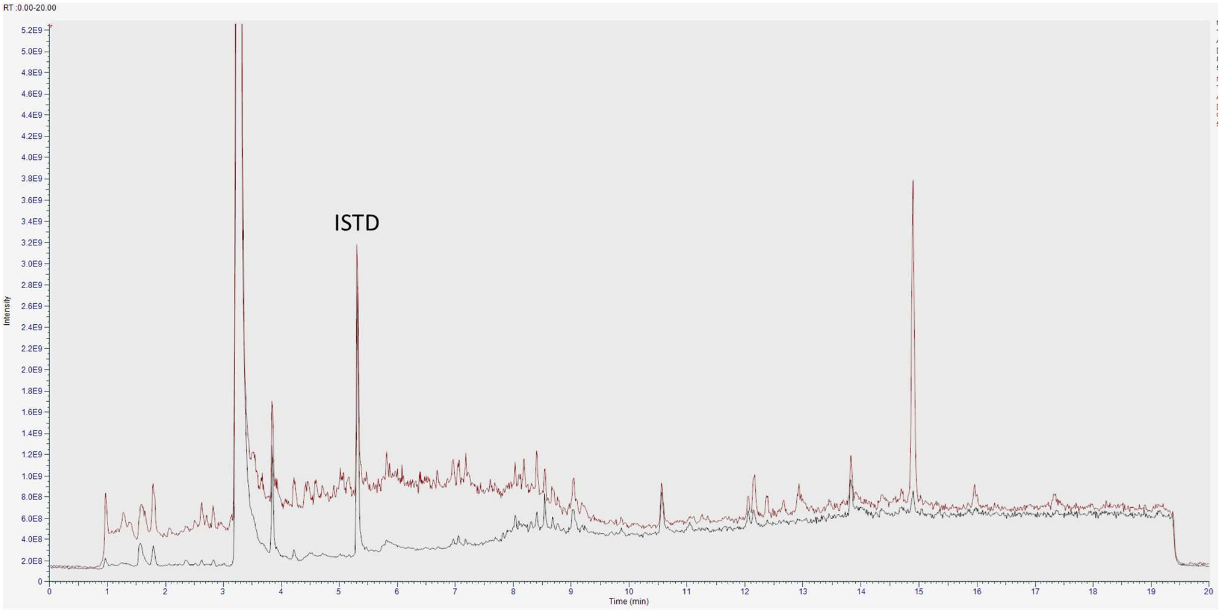

Fig. S2 LC-HRAM-MS total ion chromatograms of THS aerosol (black trace) and CC smoke (red trace) acquired with the RP-APCI(+) method; "ISTD" denotes the peak of the internal standard.

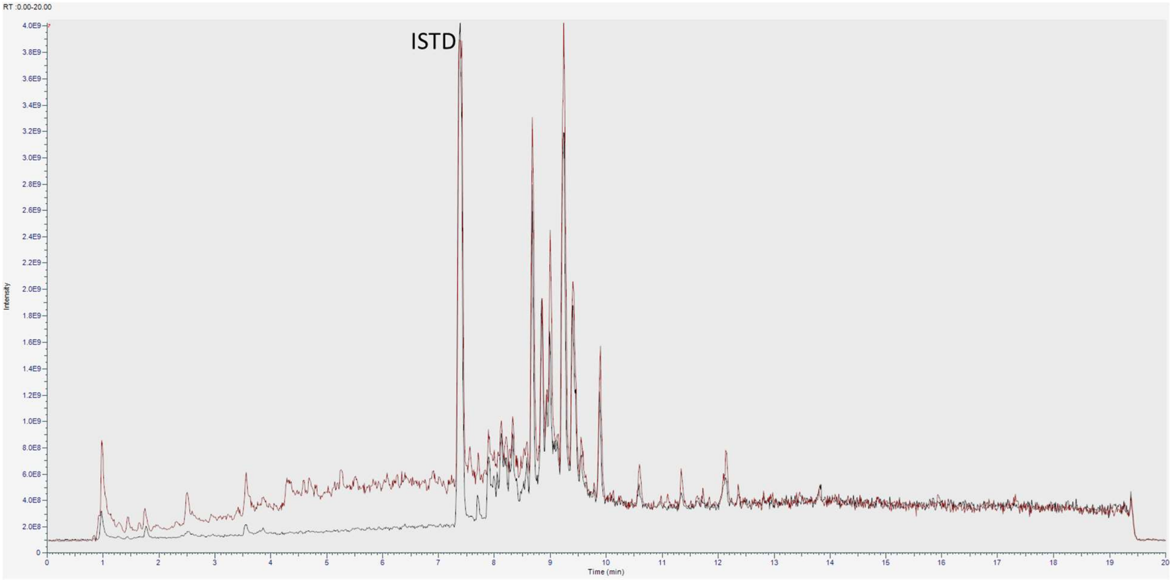

Fig. S3 LC-HRAM-MS total ion chromatograms of THS aerosol (black trace) and CC smoke (red trace) acquired with the RP-HESI(-) method; “ISTD” denotes the peak of the internal standard.

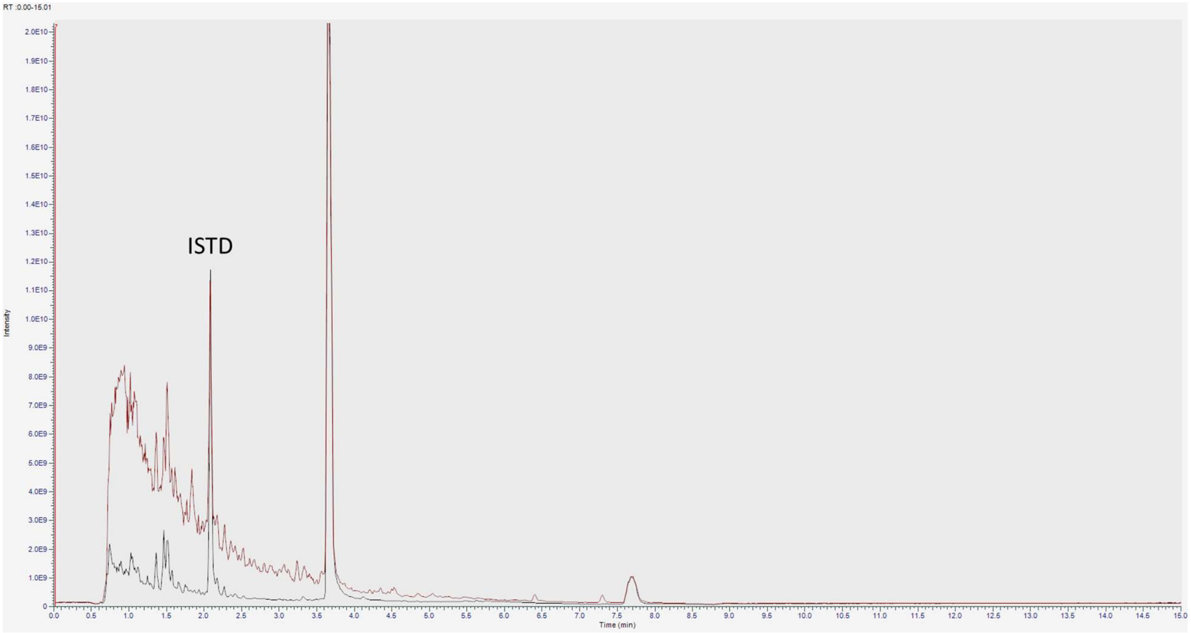

Fig. S4 LC-HRAM-MS total ion chromatograms of THS aerosol (black trace) and CC smoke (red trace) acquired with the HILIC-HESI(+) method; “ISTD” denotes the peak of the internal standard.

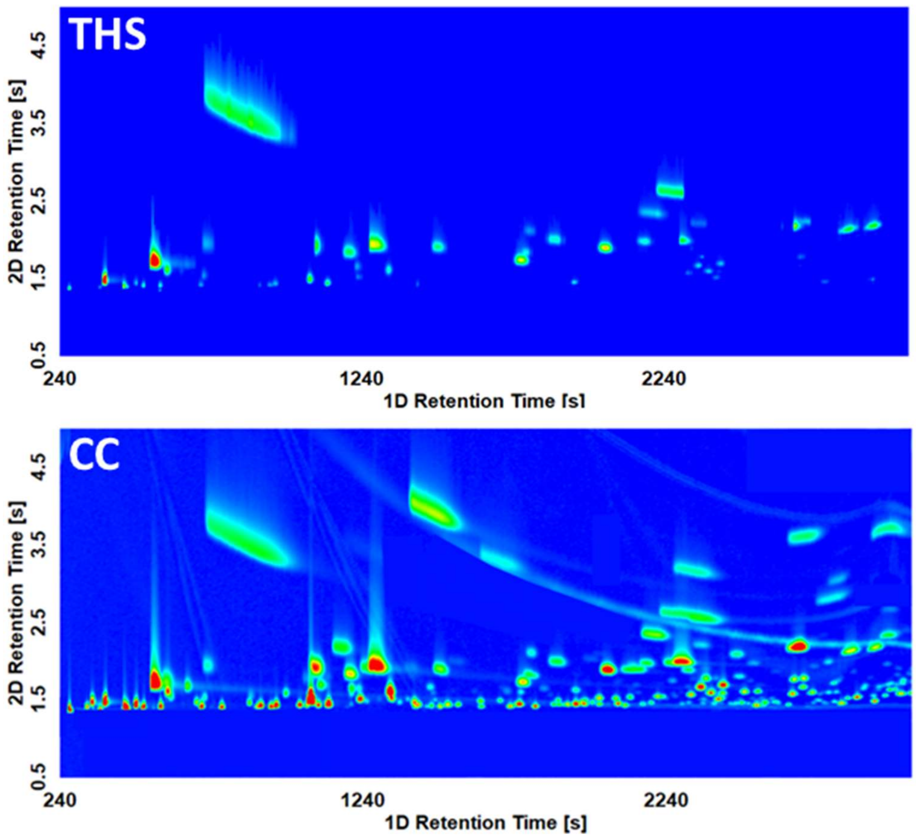

Fig. S5 GC×GC-TOFMS apex ion chromatograms of THS aerosol and CC smoke acquired with the Volatile method; both chromatograms are displayed with the same intensity range. Internal standards and retention index markers were removed for clarity.

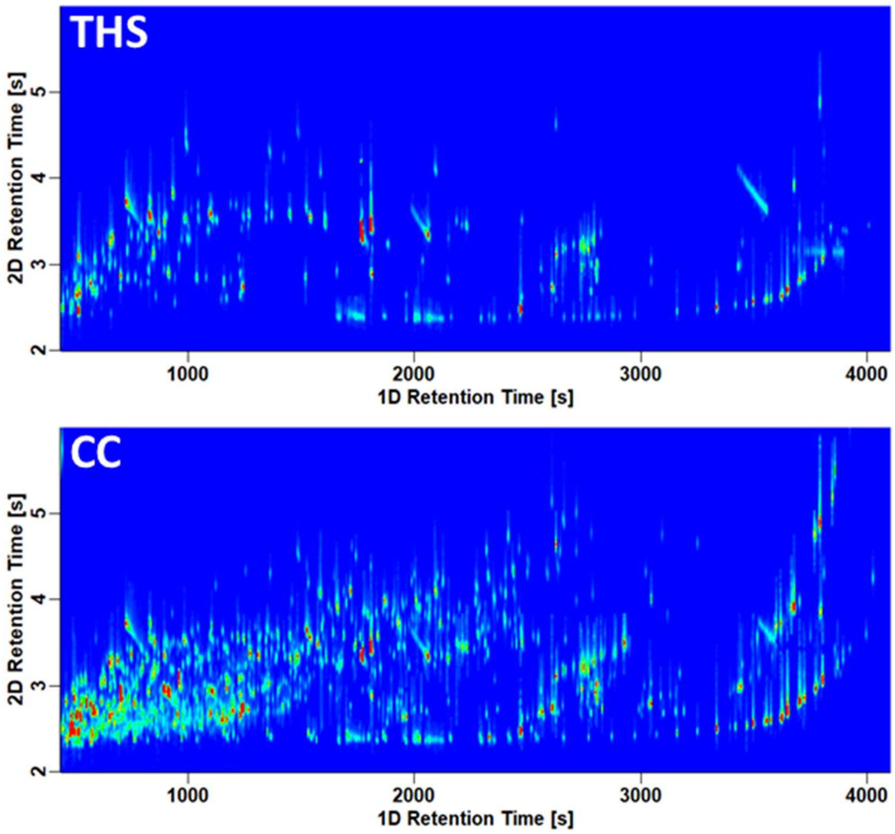

Fig. S6 GC×GC-TOFMS apex ion chromatograms of THS aerosol and CC smoke acquired with the Nonpolar method; both chromatograms are displayed with the same intensity range. Internal standards and retention index markers were removed for clarity.

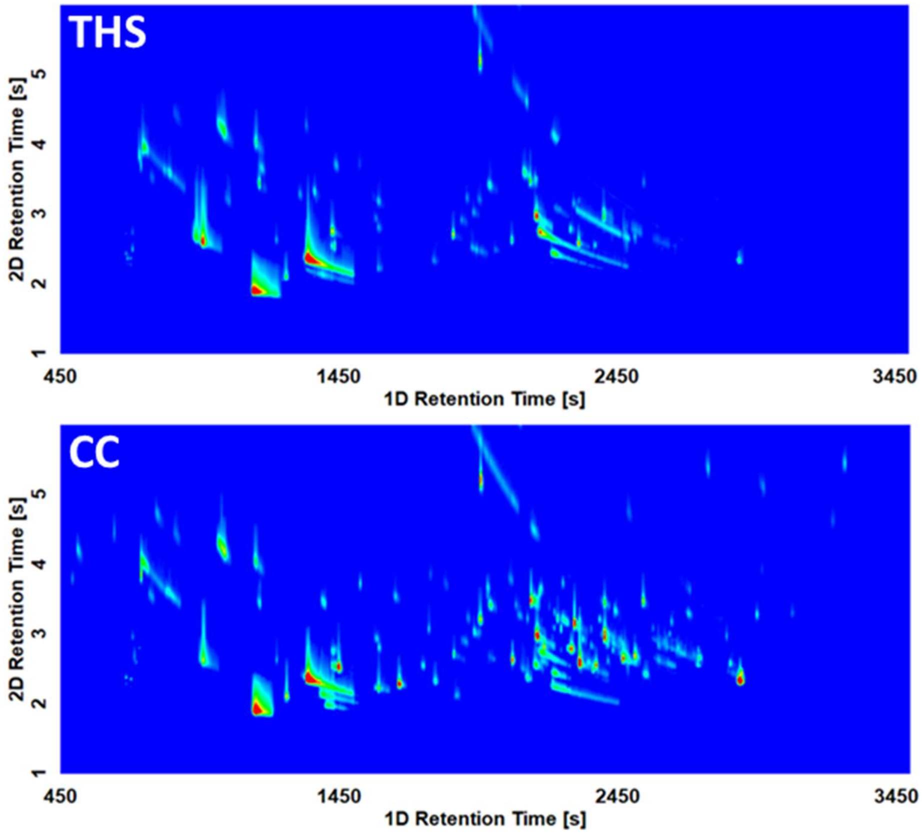

Fig. S7 GC×GC-TOFMS apex ion chromatograms of THS aerosol and CC smoke acquired with the Polar method; both chromatograms are displayed with the same intensity range. Internal standards and retention index markers were removed for clarity.
